# Focus on Prevention: Peripheral Arterial Disease and the Central Role of the Cardiologist

**DOI:** 10.3390/jcm12134338

**Published:** 2023-06-28

**Authors:** Vincenzo Fioretti, Donato Gerardi, Giuseppe Giugliano, Aldo Di Fazio, Eugenio Stabile

**Affiliations:** 1 Division of Cardiology, Cardiovascular Department, Azienda Ospedaliera Regionale “San Carlo”, 85100 Potenza, Italy; 2 Department of Advanced Biomedical Sciences, Federico II University of Naples, 80131 Naples, Italy; 3 Regional Complex Intercompany Institute of Legal Medicine, Azienda Ospedaliera Regionale “San Carlo”, 85100 Potenza, Italy; aldo.difazio@ospedalesancarlo.it

**Keywords:** peripheral artery disease, atherosclerosis, cardiologist, prevention

## Abstract

Peripheral artery disease (PAD) is a manifestation of systemic atherosclerotic disease. PAD patients have a poor prognosis with an increased risk of cardiovascular (CV) events, including myocardial infarction (MI), stroke, limb ischemia and CV death; therefore, it is important to detect and treat PAD early. PAD and coronary artery disease (CAD) share a common pathogenesis and risk factors for development; therefore, cardiologists are in a unique position to screen, diagnosis and treat PAD. Moreover, PAD and CAD also share some treatment goals, including an aggressive modification of risk factors to reduce the risk of CV events. However, PAD remains an underdiagnosed and undertreated disease with medico-legal implications. As the role of cardiologists is expanding, the purpose of this review was to awaken the clinicians to the significance of PAD.

## 1. Introduction

Atherosclerosis is a systemic, inflammatory vascular disorder frequently involving one or more vascular beds in the same subject [1]. The term peripheral arterial disease (PAD) refers to a broad spectrum of clinical manifestations that can affect several vascular beds, including upper and lower extremities, and carotid, vertebral, mesenteric and renal arteries; this review focuses on atherosclerosis of the lower extremities. PAD patients have a high risk of all-cause and CV mortality, and a higher risk of stroke or myocardial infarction (MI), at least equivalent to the risk in patients with coronary artery disease (CAD) [2]. Due to the multisite localization of atherosclerotic arterial disease, cardiologists are in a unique position to screen, diagnose and treat PAD [3]. The following review discusses the incidence and diagnosis of multisite arterial disease and the available medical management strategies to improve outcomes in patients with lower extremity peripheral arterial disease (LE-PAD).

## 2. Pathophysiology Background

LE-PAD refers to the acute or chronic obstruction of the arteries supplying the lower extremities, resulting in reduced blood flow, that is responsible for the different clinical manifestations from intermittent claudication to critical limb ischemia (CLI). Most often, the underlying disease process is atherosclerosis, a chronic disease characterized by lipid accumulation in the intima layer and subsequent inflammation. Atherosclerosis often coexists with arteriosclerosis, which is characterized by the stiffening and thickening of the arterial wall, involving degenerative changes in the extra-cellular matrix of the media layer. Moreover, arteriosclerosis is an independent predictor of morbidity and all-cause mortality [4].

The well-known modifiable risk factors associated with atherosclerotic CAD and carotid occlusive disease (COD) also contribute to atherosclerosis of the lower limb arteries. The risk factors more strongly associated with the greatest risk of LE-PAD are cigarette smoking and diabetes mellitus; other risk factors include dyslipidemia, hypertension, chronic kidney disease, obesity and inflammation, as measured by C-reactive protein concentration [5]. On the contrary, arterial stiffening is strongly associated with age and blood pressure [6]. LE-PAD may less frequently result from thrombosis, embolism, vasculitis, fibromuscular dysplasia, or entrapment.

## 3. Epidemiology and Prognosis

### 3.1. CAD in Patients with LE-PAD

**Figure 1:**
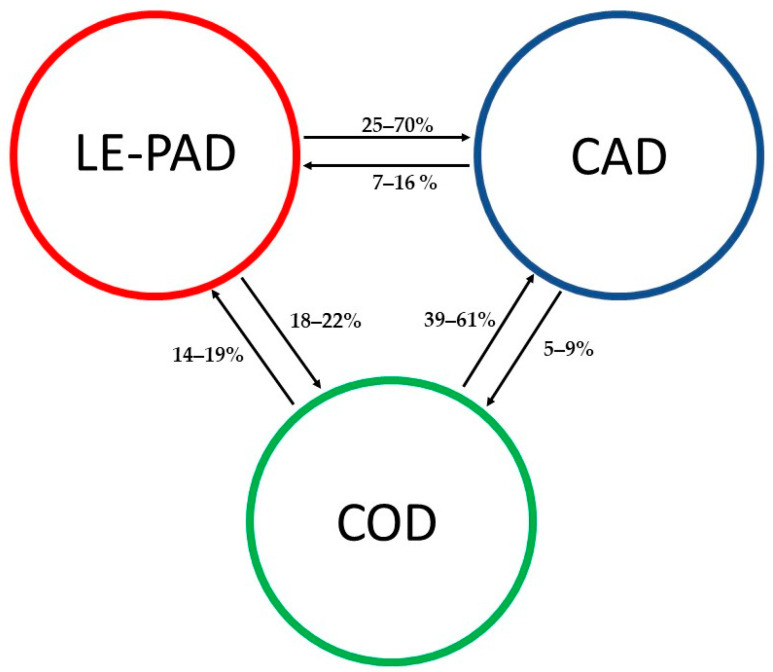
Multisite artery disease and ranges of other localization of atherosclerosis in patients with a specific arterial disease: LE-PAD = lower extremity peripheral arterial disease; CAD = coronary artery disease; COD = carotid occlusive disease (severe carotid artery stenosis ≥ 70%).

LE-PAD affects more than 200 million people worldwide [7]. Following CAD and stroke, LE-PAD is the third most frequent form of atherosclerotic CV disease. The European Society of Cardiology (ESC) estimated that in 2019 about 29.5 million people lived with LE-PAD across 57 European member countries [8]. The prevalence of LE-PAD increases significantly with advancing age: the worldwide prevalence in individuals aged 60–64 years is about 8% and progressively increases to about 25% among individuals ≥90 years [5]. In the PESA (Progression of Early Subclinical Atherosclerosis) study, the iliofemoral territory was the most frequently affected vascular site in middle-aged asymptomatic participants. Furthermore, having atherosclerotic disease in the iliofemoral district determines a 70% probability of finding disease in any other arterial territory. Conversely, the absence of disease in the iliofemoral district is associated with a 67% probability of being disease-free in the other vascular territories [9]. The simultaneous presence of atherosclerotic disease in at least two major vascular territories is defined as “multisite” artery disease [10]. Patients with LE-PAD have a higher risk of subclinical coronary and cerebrovascular disease and are at a higher risk of CV events than healthy controls [11]. According to the data reported in the current ESC guidelines, the prevalence of angiographically significant CAD in patients with LE-PAD ranges between 25% and 70% (Figure 1) [12]. In the REACH (Reduction of Atherothrombosis for Continued Health) registry, almost two-thirds of LE-PAD patients had a coexisting CAD or cerebrovascular disease [13,14]. An abnormal ankle brachial index (ABI) was associated with an increased risk of CV and all-cause mortality: in patients with an ABI between 0.81 and 0.90, the total mortality was doubled, and in those with an ABI ≤ 0.70, it was quadrupled [15]. The specificity of an abnormal ABI in predicting future CV events is approximately 90% [16]. The risk of CV mortality increased along with the severity of the LE-PAD stage [17]. In a Swedish observational study, the Cox proportional hazard model revealed an almost doubled 10-year risk of CV mortality in asymptomatic patients (HR 1.9), an HR of 2.6 in patients with intermittent claudication (IC) and an HR of 3.5 in patients with severe limb ischemia [18]. LE-PAD and its severity also predict the extension and complexity of CAD: ABI can be employed as a useful predictor for CAD complexity assessed by SYNTAX Score [19], and a higher Trans-Atlantic Inter-Society Consensus II classification is also associated with a higher SYNTAX Score [20].

### 3.2. LE-PAD in Patients with CAD

CAD is the most common type of heart disease and is a leading cause of death globally [21]. Similar to LE-PAD, CAD prevalence increases with age, and the lifetime risk of developing CAD in men and women after 40 years of age is 49% and 32%, respectively [22]. The REACH dataset found that almost one-third of the patients with CAD also presented LE-PAD [13,14]. Similarly, the GenePAD study also reported that almost one-fourth of the patients with CAD had a concomitant LE-PAD, supporting a common pathophysiology [23]. According to the data reported in the current ESC guidelines, the prevalence of LE-PAD in patients with CAD ranges between 7% and 16% (Figure 1) [12]. Moreover, in patients admitted for CAD, LE-PAD negatively influenced the prognosis. The presence of LE-PAD is a variable considered in Syntax Score II to predict mortality in the decision-making of the heart-team between coronary artery bypass grafting or percutaneous coronary intervention (PCI) in patients with left-main or three-vessel coronary artery disease [24]. In patients with MI, the presence of concomitant PAD is associated with an increased risk of adverse events. In a large analysis of 2 million patients from the National Inpatient Sample presenting with MI, approximately half of the population had pre-existent PAD and an incremental increase in adverse outcomes with an increasing number of vascular beds. Among those with a single pre-existent diseased vascular bed, the incidence of adverse events was higher in those with LE-PAD [25]. Moreover, in a large analysis of 1.4 million patients from the National Inpatient Sample undergoing PCI, the overall prevalence of PAD was 14%, and all-cause mortality was 22% higher in patients with PAD compared to those without. However, the strength of this association varied, with LE-PAD having the greatest impact on mortality, followed by cerebrovascular disease. In addition, for MI, the odds of in-hospital mortality increased progressively with the number of vascular beds involved [26]. Therefore, due to the close relationship between CAD and PAD, it is important to suspect and detect these conditions early.

### 3.3. COD in Patients with LE-PAD

The term “carotid occlusive disease” usually refers to atherosclerotic disease involving the extracranial internal carotid artery. The prevalence of COD is slightly higher in men and increases significantly with advancing age. For men, the prevalence of moderate carotid artery stenosis (degree of stenosis ≥ 50%) increases from 0% to 7.5% with advancing age, while the prevalence of severe carotid artery stenosis ranges from 0.1% to 3.1% [27]. Stroke is the second leading cause of both disability and death worldwide. COD is a major cause of cerebral ischemic events, accounting for approximately 10–20% of ischemic strokes [28]. Due to the same pathogenesis, COD is frequent in patients with LE-PAD. According to the data reported in the current ESC guidelines, the prevalence of severe COD (degree of stenosis ≥ 70%) in patients with LE-PAD ranges between 14% and 19% (Figure 1) [12]. The prevalence of significant COD (degree of stenosis ≥ 50%) increases with the severity of LE-PAD: ABIs less than 0.5 and Fontaine Stage IV were reported as independent risk factors for significant COD [29].

### 3.4. LE-PAD in Patients with COD

Very few data exist regarding the prevalence of LE-PAD in patients with carotid artery disease. According to the data reported in the current ESC guidelines, the prevalence of LE-PAD in subjects with severe COD ranges between 18 and 22% (Figure 1) [12]. A significant prevalence of LE-PAD in patients with cerebrovascular events has been reported in some studies, and the severity of LE-PAD is related with an increased risk of recurrent cerebrovascular events. In the OECROSS study involving patients with ischemic stroke or TIA, the prevalence of LE-PAD (ABI ≤ 0.9) was 45%; an ABI ≤ 0.9 was significantly related to the presence of significant COD, so LE-PAD can contribute to the risk stratification of these patients [30]. In the ARTICO study, in patients with a first noncardioembolic stroke, an increased risk of recurrent vascular events at follow-up was significantly associated with symptomatic LE-PAD [31].

## 4. Diagnosis

Multisite artery disease is invariably associated with worse clinical outcomes [32]; however, screening for asymptomatic disease in other sites has not been proven to improve prognosis. The Active Detection and Management of the Extension of Atherothrombosis in High-Risk Coronary Patients in Comparison with Standard of Care for Coronary Atherosclerosis (AMERICA) study evaluated in high-risk coronary patients a proactive strategy for the detection of asymptomatic extra-coronary atherothrombotic disease combined with an aggressive pharmacological secondary prevention strategy and/or revascularization (proactive strategy) as compared with a conservative strategy based on a clinically guided identification of multisite artery disease and standard pharmacological treatment (conventional strategy). At a two-year follow-up, the proactive strategy did not reduce the rate of CV events compared with the conventional strategy; although there was a low rate of revascularizations in the proactive strategy group, an aggressive secondary prevention was performed in both groups, limiting the impact of the proactive strategy [33]. Therefore, in patients with any presentation of PAD, as suggested by ESC guidelines, a clinical assessment of symptoms and signs of disease in other localizations and/or CAD is necessary, and in case of clinical suspicion, further tests may be planned [12].

### 4.1. LE-PAD Diagnosis

During a cardiology visit, it is important to investigate symptoms related to LE-PAD, in particular, limb pain either with exercise (intermittent claudication) or at rest. A physical examination should include palpation of the peripheral pulses, inspection of the extremities and auscultation of accessible arteries for bruits. The most used classifications of LE-PAD are Fontaine and Rutherford. The ankle brachial index (ABI) is a simple bedside tool to screen and diagnose LE-PAD. This index is the ratio of systolic blood pressure (SBP) measured at the ankle to SBP measured at the brachial artery. The normal ABI range is 1.00 to 1.40. An ABI value to 0.91 to 0.99 is borderline, and an ABI of 0.90 or less is abnormal. An ABI of 0.90 or lower has a specificity of 83% to 99% and a sensitivity of 69% to 73% in detecting stenosis greater than 50%. Patients with symptoms of leg claudication often have an ABI ranging from 0.5 to 0.8, and patients with CLI usually have an ABI lower than 0.5. In patients with diabetes or renal insufficiency, ABI is often falsely high, due to medial artery calcification. In this scenario, it is recommended to measure the toe brachial index (TBI), the ratio of toe to brachial SBP, because medial calcification rarely affects digital arteries. A TBI < 0.7 is generally considered to be abnormal. ABI is also one of the screening tests able to detect hypertension-mediated organ damage (HMOD). However, in the community-based Framingham Study, a low ABI was the least prevalent (<5%), while an elevated carotid–femoral pulse wave velocity was the most prevalent HMOD (40–60%). Left ventricular hypertrophy, reduced kidney function, microalbuminuria, increased carotid intima-media thickness and abnormal brain imaging findings had an intermediate prevalence (20–40%) [34]. Different from ABI, that is, linked to atherosclerotic disease, pulse wave velocity (PWV) is the most validated method to noninvasively quantify arterial stiffness. PWV is inversely related to vascular compliance. Hence, a stiffer vessel will conduct the pulse wave faster than a more compliant vessel. Lower-limb arterial stiffness can be determined using femoral–ankle pulse wave velocity (faPWV). Both ABI and faPWV are independently associated with CV disease [35]. A variety of noninvasive imaging tests are available to detect LE-PAD and to characterize the severity of the disease. Duplex ultrasound is an accessible and reliable method; its sensitivity and specificity depend on several factors, including the presence of calcium in the arterial wall, the location or depth of the vessel and the presence of multiple occlusions at different locations. Other noninvasive imaging modalities include magnetic resonance angiography (MRA) and computed tomography (CT) angiography, which provide high-resolution images but at the price of radiation exposure and the need for contrast agents. Angiography is the diagnostic gold standard and is typically reserved for patients who also need concomitant endovascular revascularization.

ESC guidelines recommend to screen for LE-PAD at least by clinical examination and/or ABI for the following conditions: patients aged > 65 years, high CV risk, evidence of atherosclerosis in other sites and patients undergoing coronary artery bypass grafting (CABG) requiring saphenous vein harvesting [12].

### 4.2. CAD Diagnosis

Due to the frequent coexistence of PAD and CAD, in patients with LE-PAD, it is important to investigate symptoms related to CAD, such as angina pectoris and anginal equivalents such as dyspnea, although LE-PAD, due to physical activity limitation, could mask these symptoms. A resting 12-lead ECG is recommended as a first-line exam in patients with suspected CAD. The most common abnormalities on the ECG are nonspecific ST-T wave changes with or without abnormal Q waves. A resting transthoracic echocardiogram is recommended in all patients for the exclusion of alternative causes of angina, identification of regional wall motion abnormalities suggestive of CAD, and measurement of left ventricular ejection fraction (LVEF) for risk stratification and the evaluation of diastolic function. Based on the pre-test probability of obstructive CAD, according to age, sex and the nature of the symptoms, either a functional (stress echocardiography, SPECT, PET, CMR, exercise ECG) or anatomical (coronary computed tomography angiography or invasive coronary angiography) test can be used to establish a diagnosis of obstructive CAD [36]. ESC guidelines recommend screening with ECG in patients with LE-PAD and with imaging stress testing in patients with poor functional capacity and more than two of the following: history of CAD, heart failure, stroke or transient ischemic attack (TIA); chronic kidney disease (CKD); or diabetes mellitus requiring insulin therapy (Class I) [12].

### 4.3. COD Diagnosis

A physical examination during a cardiology visit should include carotid artery auscultation to detect carotid bruit. A variety of noninvasive imaging tests are available to detect COD and to characterize the severity of the disease. Duplex ultrasound combining B-mode anatomical imaging with Doppler flow velocity characteristics can be used to determine the presence of atherosclerosis and the flow status of the carotid artery. Duplex ultrasound is considered the first-line carotid imaging modality to assess extracranial carotid stenoses. Other noninvasive imaging modalities include MRA and CT angiography, which provide images from the aortic arch up to the intracranial circulation, as well as brain parenchyma. Intra-arterial digital subtraction angiography is rarely required for diagnostic purposes. In the current ESC guidelines, there are no specific recommendations regarding the screening of COD in patients with LE-PAD [12].

## 5. Treatment

Atherosclerosis is the main cause of PAD, and the modifiable risk factors are not different from patients with coronary artery disease. The treatment of LE-PAD aims to reduce CV morbidity and mortality, as well as improve quality of life by decreasing symptoms of claudication, eliminating rest pain and preserving limb viability [12]. Therapeutic considerations therefore include the aggressive modification of risk factors by modifications in lifestyle and the use of pharmacologic therapy to reduce the risk of adverse CV events such as MI, stroke and death, as well as to decrease limb morbidity.

### 5.1. Lifestyle Modifications

Lifestyle modifications include healthy diet, weight loss, regular physical exercise and smoking cessation [27]. Adherence to a healthy diet is associated with a lower incidence of clinical PAD; The Mediterranean diet was associated with a reduced risk of PAD [37]. Physical activity, especially low- to moderate-intensity aerobic exercise, is associated with a decrease in CV mortality, as well as the risk of developing CV disease [38]. Moreover, supervised exercise therapy is an effective strategy to reduce claudication symptoms and improve functional outcomes and is recommended for the treatment of symptomatic LE-PAD [39]. Tobacco exposure, through cigarette smoking, is strongly associated with the development and progression of LE-PAD and its complications [40]. Smoking cessation is associated with a decreased risk of major adverse cardiac events (MACEs) and major adverse limb events (MALEs) [41]. Further, patients who stop smoking have improved walking ability and decreased claudication symptoms [42]. The management of smoking cessation includes behavioral counseling and pharmacological therapy including nicotine replacement therapy, bupropion and varenicline.

### 5.2. Treatment of Diabetes Mellitus

Diabetes mellitus (DM) is one of the strongest predictors for PAD. Patients with DM have an approximately two-fold increased risk of all-cause mortality than those without diabetes [43]. Moreover, DM is associated with an increased risk of amputation in LE-PAD patients [44]. The aggressive treatment of DM decreases the risk of microangiopathic events, but most classes of glucose-lowering drugs have not shown a reduction in macrovascular events. The data to support glycemic control to improve outcomes in patients with PAD and DM are conflicting. The long-term follow-up of the UKPDS (United Kingdom Prospective Diabetes Study) of patients with type 2 DM found that intensive treatment was associated with a 15% reduction in MI, suggesting a positive glycemic legacy in patients with newly diagnosed DM and without prior CV events [45]. Other studies based on an intensive glucose-lowering strategy demonstrated no benefit for MACE reduction [46,47]. Newer diabetes therapies have demonstrated large benefits for patients with DM that cannot be explained by glycemic control alone. There is now growing evidence to support the use of sodium–glucose cotransporter inhibitors (SGLT-2is) in patients with PAD and diabetes [48]. The EMPA-REG OUTCOME trial demonstrated that empaglifozin reduced the risk of CV death, hospitalizations for heart failure and the progression of renal disease with no observed increase in the risk of lower limb amputation in patients with PAD [49]. A secondary analysis from the DECLARE-TIMI 58 trial demonstrated that dapaglifozin reduced the risk of CV death, hospital admissions for heart failure and the progression of kidney disease with no significant differences in any limb outcome versus the placebo in patients with PAD [50]. Instead, in the Canagliflozin Cardiovascular Assessment Study (CANVAS), canagliflozin was associated with an increased risk of lower limb amputation [51]. However, this result was not confirmed in the Canagliflozin and Renal Events in Diabetes with Established Nephropathy Clinical Evaluation (CREDENCE) trial [52].

In addition, glucagon-like peptide 1 receptor agonists (GLP-1RAs), due to their pleiotropic effects, are emerging drugs in the treatment of diabetes to reduce the CV risk [53]. In a post hoc analysis of the Liraglutide Effect and Action in Diabetes: Evaluation of Cardiovascular Outcome Results (LEADER) trial, the treatment with liraglutide in patients with type 2 diabetes and a high risk of CV events was associated with a significantly lower risk of amputations compared with the placebo [54]. In a recent observational study, the use of GLP-1RAs was associated with significantly lower risks of MALEs when compared with the use of dipeptidyl peptidase 4 inhibitors (DPP4is). The risk reduction was driven largely by a reduced rate of amputations. Moreover, the treatment with GLP-1RAs was also associated with lower risks of CV death, nonfatal stroke, nonfatal MI and death from any cause [55]. Two recent meta-analyses demonstrated a lower incidence of lower limb amputations in patients receiving GLP-1RAs in comparison with patients receiving SGLT-2is [56,57]. In contrast, a meta-analysis comparing the impact of GLP1-RAs and DPP4is versus SGLT-2is showed the incretin-based therapies had a 10% higher risk of lower limb amputation compared to the SGLT-2i group [58]. Further randomized controlled trials are needed to assess the impact of these antidiabetic drugs on lower-limb-related events.

ESC guidelines recommend SGLT2 inhibitors and GLP-1RAs in patients with type 2 diabetes mellitus and CV disease or a very high/high CV risk to reduce CV events (Class I A). An HbA1c of <7.0% (<53 mmol/mol) is recommended to decrease microvascular complications in individuals with diabetes (Class I A), while the same target should be considered for the prevention of macrovascular complications (Class IIa C) [59].

### 5.3. Treatment of Hypertension

Hypertension is a common and important risk factor for PAD. An analysis of 4.2 million relatively healthy adults showed that a 20 mm Hg higher than usual systolic blood pressure was associated with a 63% higher risk of peripheral arterial disease [60].

Diuretics, beta-blockers, calcium antagonists, angiotensin-converting enzyme inhibitors (ACEIs) and angiotensin receptor blockers (ARBs) can be used to treat high blood pressure, as a monotherapy or in different combinations [61]. The Heart Outcomes Prevention Trial (HOPE) and the Ongoing Telmisartan Alone and in Combination With Ramipril Global Endpoint Trial (ONTARGET) have shown that ACEIs and ARBs significantly reduce CV events in patients with PAD [62,63]. Moreover, ACEIs improved walking ability in patients with intermittent claudication [64] and are associated with improved amputation-free survival in patients undergoing peripheral vascular intervention for chronic limb-threatening ischemia [65]. Since beta-blockers have not been shown to worsen the symptoms of claudication [66], they remain a treatment option in hypertensive patients with LE-PAD. ESC guidelines recommend that ACEIs and ARBs should be considered as the first-line therapy in patients with PAD and hypertension (Class IIa B), with a primary goal of a blood pressure less than 140/90 mmHg (Class I A) [12] and with specific targets according to the risk factors and associated diseases [61].

### 5.4. Lipid-Lowering Treatment

Dyslipidemia is a key pathogenic factor predisposing to atherosclerosis. Patients with PAD, according to the levels of CV risk proposed by guidelines, should be considered to have a very high CV risk, such as patients with previous acute coronary syndrome. Therefore, in the secondary prevention for patients with very high risk, an LDL-C reduction of ≥50% from the baseline and an LDL-C goal of <1.4 mmol/L (<55 mg/dL) are recommended [67]. Different drugs for the treatment of dyslipidemia are available, including statins, ezetimibe, proprotein convertase subtilisin/kexin type 9 (PCSK9) inhibitors (alirocumab and evolocumab), PCSK9 synthesis inhibitor (inclisiran) and adenosine triphosphate–citrate lyase inhibitor (bempedoic acid) [68]. Statins represent the first-line treatment and their efficacy in PAD patients, derived from the UK Heart Protection study, was demonstrated in the efficacy of a therapy with 40 mg simvastatin in reducing the incidence of MACEs by 22% compared with the placebo and reducing the risk of the first acute peripheral vascular event (noncoronary revascularization, major amputation, aneurysm repair or death due to PAD) by 16% [69]. In a recent meta-analysis including 138,060 patients with PAD, the use of statins was associated with a 30% reduction in MALEs and a 35% reduction in amputations. The statin group also had a lower risk of all-cause mortality, CV death, composite CV endpoints and ischemic stroke [70]. The addition of ezetimibe to statins was associated with an 8% relative risk reduction in CV death, major coronary events and stroke compared to statins alone in patients with acute coronary syndrome [71], and this benefit was also confirmed in patients with concomitant polyvascular disease [72]. Confirming the benefit of treatments that reduce plasma LDL levels, the FOURIER trial showed that evolocumab significantly reduced the risk of CV death, MI, stroke and hospitalization for unstable angina or coronary revascularization by 15% and reduced the risk of MALEs by 37% [73]. Moreover, alirocumab in the ODYSSEY OUTCOMES trial reduced the risk of death related to CAD, nonfatal MI, ischemic stroke or hospitalization for unstable angina by 15% and reduced the risk of PAD events by 31% [74]. Despite the promising results in patients with high CV risk [75,76], the effects of inclisiran and bempedoic acid in PAD remain to be explored.

### 5.5. Antithrombotic Treatment

Antiplatelet therapy remains a key intervention to reduce CV risk in PAD. Antithrombotic treatment is different according to the symptomatic status, history of revascularization and type of revascularization [12].

In *asymptomatic patients* with a low ABI but without clinical limb symptoms or previous vascular intervention, although at an increased risk of MACEs and MALEs, antiplatelet therapy is not recommended [77,78].

In *symptomatic patients* with intermittent claudication and without previous vascular intervention, antiplatelet drugs improve CV prognosis; therefore, ESC guidelines recommend long-term single antiplatelet therapy, preferring clopidogrel to aspirin [12]. The CAPRIE trial (Clopidogrel versus Aspirin in Patients at Risk for Ischemic Events) demonstrated that patients randomized to 75 mg of clopidogrel daily had a significant reduction in MACEs compared with patients randomized to 325 mg of aspirin [79]. Single-antiplatelet therapy with ticagrelor and dual-antiplatelet therapy (DAPT) with aspirin and clopidogrel provided no benefit in this group of patients [80,81]. A consensus document from the ESC working group on aorta and peripheral disease included recommendations about low-dose rivaroxaban, an oral inhibitor of Xa factor, for patients with a low bleeding risk [82]. The COMPASS trial showed that dual-pathway inhibition (DPI) with low-dose rivaroxaban (2.5 mg twice daily) in addition to 100 mg of aspirin daily reduced the risk of MACEs and MALEs compared with aspirin alone in patients with LE-PAD or significant carotid artery disease, as well as in those with symptomatic LE-PAD. However, the DPI regimen was associated with an increased risk of major bleeding, without increasing the risk for fatal or intracranial bleeding [83].

In patients undergoing endovascular revascularization, guidelines recommend DAPT with aspirin and clopidogrel for at least one month [12]; however, the choice, dose and duration of antithrombotic drug therapy in relation to endovascular procedures is unclear. In the MIRROR study (Management of Peripheral Arterial Interventions with Mono- or Dual-Antiplatelet Therapy), patients with LE-PAD after endovascular interventions were randomized to receive either aspirin or aspirin and clopidogrel for 6 months. At 6 months, there was a significant reduction in the need for target lesion revascularization (TLR) in the DAPT group compared to the aspirin monotherapy. All patients discontinued clopidogrel at 6 months and continued aspirin monotherapy per the study protocol. At 12 months, similar TLR rates were observed in patients who had received the DAPT or aspirin monotherapy [84].

In patients undergoing open revascularization, guidelines recommend single-antiplatelet therapy, but there is no robust evidence for which antithrombotic strategy is most effective to maintain vein graft patency [12]. Anticoagulation with vitamin K antagonists is recommended in patients who receive infrainguinal bypass using an autologous vein conduit with high-risk features, including poor quality conduit, long conduit, disadvantaged distal runoff or previous failed open revascularization [85]. The CASPAR trial showed no benefit of DAPT over single-antiplatelet therapy in patients undergoing below-knee bypass grafts, but in a subgroup analysis, DAPT conferred benefit in patients receiving prosthetic grafts without significantly increasing major bleeding risk [86].

The consensus document of the ESC working group on aorta and peripheral disease also included recommendations regarding DPI in patients undergoing revascularization [82]. The VOYAGER PAD trial randomized patients after endovascular or open revascularization to a combination therapy of rivaroxaban (2.5 mg twice daily) and aspirin or aspirin alone, with the possibility to add clopidogrel up to a maximum of 6 months at the treating physicians’ discretion. Compared with aspirin alone, the combination of rivaroxaban and aspirin reduced the composite endpoint of MACEs and MALEs, largely driven by a significant reduction in acute limb ischemia. Although there was no significant difference in the primary safety outcome of Thrombolysis in Myocardial Infarction (TIMI) major bleeding, the secondary safety outcome of the International Society on Thrombosis and Haemostasis major bleeding was increased, albeit without significant increases in intracranial or fatal bleeding. Approximately one-half of the VOYAGER trial participants were given clopidogrel. The mean duration of clopidogrel use was 30 days, and the use of concomitant clopidogrel after revascularization did not alter the efficacy of DPI compared with aspirin alone in reducing MACEs or MALEs [87]. However, in those with longer courses of clopidogrel, there was a trend toward increased major bleeding [88].

## 6. Medico-Legal Implications

The failure to diagnose LE-PAD can have serious and devastating consequences for the patient. Most civil litigation cases concern patients with critical limb ischemia, which is considered the final stage of LE-PAD. The failure to promptly treat this pathological condition leads to the unnecessary amputation of the affected limb with the related morbidity and mortality burden.

To avoid succumbing in any cases of judicial litigation, the physician must demonstrate that they have followed either the available guidelines or good clinical practices for the diagnosis and treatment of this disease. Therefore, it is essential at the time of the first contact between the physician and the patient to document the signs and symptoms, for example, by highlighting whether LE-PAD is associated to claudication, pain at rest or the presence of trophic lesions. In case of the suspicion of critical limb ischemia, it will be necessary to request the advice of a vascular specialist, which must be performed as soon as possible since this clinical condition is considered a vascular emergency. From a medico-legal point of view, in fact, the most recurring critical issues concern the failure to recognize the clinical signs of this pathological condition and the inadequate period that elapses between the manifestation of the clinical conditions and the request for an accurate assessment by a vascular specialist. Further claims for damages concern mistakes in postoperative monitoring and an adequate acquisition of informed consent. Regarding this last aspect, the informed consent must explain what the therapeutic procedure consists of, the generic and specific risks related to it (preferably indicating the percentages of occurrence of the individual events), any therapeutic alternatives and the risks deriving from any refusal to undergo the proposed intervention.

## 7. Discussion

The incidence of LE-PAD continues to increase, and it is estimated that it affects more than 200 million people worldwide [7]. LE-PAD correlates strongly with the risk of MACEs because it is frequently associated with coronary and cerebral atherosclerosis [2]. Therapeutic considerations therefore include the modification of risk factors by alterations in lifestyle and the use of pharmacologic therapy to reduce the risk of MACEs and MALEs [12]. Despite its frequency and poor prognosis, LE-PAD remains underdiagnosed and undertreated. A Greek registry recruiting 436 participants from 14 primary-care health centers revealed that the prevalence of LE-PAD was 13% (mostly asymptomatic, 11.7%), and only 5 (8.77%) of 57 patients with LE-PAD were aware of their disease [89]. LE-PAD is also underdiagnosed in patients at high risk admitted for CAD. A prospective study conducted at the University of Jordan Hospital, enrolling a total of 2120 patients referred for coronary angiography without a prior diagnosis of LE-PAD, demonstrated that the prevalence of asymptomatic LE-PAD in patients with CAD was 14.7% and was strongly associated with a higher incidence of CV risk factors, multivessel disease and left-main disease [90]. The 16-center PORTRAIT (Patient-Centered Outcomes Related to Treatment Practices in Peripheral Arterial Disease: Investigating Trajectories) registry enrolled 1275 patients with LE-PAD and showed that when patients receive a workup for LE-PAD symptoms in a specialty care setting, only 1 in 5 receive all eligible evidence-based medical-management-quality measures for PAD, with a high variability across institutions [91]. A retrospective study enrolled 739 patients with claudication or critical limb ischemia who underwent diagnostic or interventional lower-extremity angiography and evaluated the adherence to guideline-directed medical therapy (GDMT). GDMT at the baseline included the use of aspirin in 651 (88%), statin medications in 496 (67%), ACE inhibitors in 445 (60%) and smoking abstention in 528 (71%) patients. A total of 237 (32%) patients met all four guideline-recommended therapies. After the adjustment for baseline covariates, patients adhering to all four guideline-recommended therapies had decreased MACEs and MALEs compared to patients receiving less than four of the recommended therapies [92]. A recent large observational study enrolling 15,891 patients with LE-PAD undergoing peripheral vascular intervention revealed that almost one-half of patients were not on GDMT. The mortality risk was higher among patients who did not receive GDMT versus those who did (31.2% vs. 24.5%; HR: 1.37; 95% CI: 1.25–1.50; *p* < 0.001), as well as the risk of amputation (16.0% vs. 13.2%; HR: 1.20; 95% CI: 1.08–1.35; *p* < 0.001) [93]. These data highlight the need to incorporate ABI in the routine clinical practice of a number of different medical and affiliated specialists for the early diagnosis of LE-PAD, and also the need for a greater awareness for the best medical treatment.

## 8. Conclusions

Peripheral artery disease is an atherosclerosis-related disease. Due to the same underlying pathogenesis, peripheral artery disease and coronary heart disease frequently coexist; therefore, cardiologists are in a unique position to screen, diagnosis and treat LE-PAD. An aggressive risk modification to reduce the risk of CV events is the basis for the treatment of both diseases. Despite the availability of different treatments and specific guidelines, patients with peripheral artery disease are often undertreated.

## Data Availability

Not applicable.

## References

[1] Björkegren J.L., Lusis A.J. (2022). Atherosclerosis: Recent developments. Cell.

[2] Agnelli G., Belch J.J., Baumgartner I., Giovas P., Hoffmann U. (2020). Morbidity and mortality associated with atherosclerotic peripheral artery disease: A systematic review. Atherosclerosis.

[3] Tran B. (2021). Assessment and management of peripheral arterial disease: What every cardiologist should know. Heart.

[4] Scandale G., Dimitrov G., Recchia M., Carzaniga G., Perilli E., Carotta M., Catalano M. (2020). Arterial stiffness and 5-year mortality in patients with peripheral arterial disease. J. Hum. Hypertens..

[5] Song P., Rudan D., Zhu Y., Fowkes F.J.I., Rahimi K., Fowkes F.G.R., Rudan I. (2019). Global, regional, and national prevalence and risk factors for peripheral artery disease in 2015: An updated systematic review and analysis. Lancet Glob. Health.

[6] Cecelja M., Chowienczyk P. (2009). Dissociation of Aortic Pulse Wave Velocity with Risk Factors for Cardiovascular Disease Other Than Hypertension. Hypertension.

[7] Mozaffarian D., Benjamin E.J., Go A.S., Arnett D.K., Blaha M.J., Cushman M., Das S.R., de Ferranti S., Després J.-P., Fullerton H.J. (2016). Heart Disease and Stroke Statistics—2016 Update: A Report from the American Heart Association. Circulation.

[8] Timmis A., Vardas P., Townsend N., Torbica A., Katus H., De Smedt D., Gale C.P., Maggioni A.P., Petersen S.E., Huculeci R. (2022). European Society of Cardiology: Cardiovascular disease statistics 2021. Eur. Heart J..

[9] Fernández-Friera L., Peñalvo J.L., Fernández-Ortiz A., Ibañez B., López-Melgar B., Laclaustra M., Oliva B., Mocoroa A., Mendiguren J., de Vega V.M. (2015). Prevalence, Vascular Distribution, and Multiterritorial Extent of Subclinical Atherosclerosis in a Middle-Aged Cohort. Circulation.

[10] Di Noi P., Brancati M.F., Burzotta F., Trani C. (2014). Multisite artery disease: A common and challenging clinical condition calling for specific management. Future Cardiol..

[11] Valentine R.J., Verstraete R., Clagett G.P., Cohen J.C. (2000). Premature cardiovascular disease is common in relatives of patients with premature peripheral atherosclerosis. Arch. Intern. Med..

[12] Aboyans V., Ricco J.-B., Bartelink M.-L.E.L., Björck M., Brodmann M., Cohnert T., Collet J.-P., Czerny M., De Carlo M., Debus S. (2018). 2017 ESC Guidelines on the Diagnosis and Treatment of Peripheral Arterial Diseases, in collaboration with the European Society for Vascular Surgery (ESVS): Document covering atherosclerotic disease of extracranial carotid and vertebral, mesenteric, renal, upper and lower extremity arteries Endorsed by: The European Stroke Organization (ESO) The Task Force for the Diagnosis and Treatment of Peripheral Arterial Diseases of the European Society of Cardiology (ESC) and of the European Society for Vascular Surgery (ESVS). Eur. Heart J..

[13] Smolderen K.G., Bell A., Lei Y., Cohen E.A., Steg P.G., Bhatt D.L., Mahoney E.M., REACH registry investigators (2010). REACH registry investigators. One-year costs associated with cardiovascular disease in Canada: Insights from the REduction of Atherothrombosis for Continued Health (REACH) registry. Can. J. Cardiol..

[14] Smolderen K., Wang K., de Pouvourville G., Brüggenjürgen B., Röther J., Zeymer U., Parhofer K., Steg P., Bhatt D., Magnuson E. (2012). Two-year Vascular Hospitalisation Rates and Associated Costs in Patients at Risk of Atherothrombosis in France and Germany: Highest Burden for Peripheral Arterial Disease. Eur. J. Vasc. Endovasc. Surg..

[15] Fowkes F., Murray G., Butcher I., Heald C.L., Lee R.J., Chambless L.E., Folsom A.R., Hirsch A.T., Dramaix M., Debacker G. (2008). Ankle Brachial Index Combined with Framingham Risk Score to Predict Cardiovascular Events and Mortality: A meta-analysis. JAMA.

[16] Aboyans V., Criqui M.H., Abraham P., Allison M.A., Creager M.A., Diehm C., Fowkes F.G.R., Hiatt W.R., Jönsson B., Lacroix P. (2012). Measurement and Interpretation of the Ankle-Brachial Index: A Scientific Statement from the American Heart Association. Circulation.

[17] Criqui M.H., Langer R.D., Fronek A., Feigelson H.S., Klauber M.R., McCann T.J., Browner D. (1992). Mortality over a Period of 10 Years in Patients with Peripheral Arterial Disease. N. Engl. J. Med..

[18] Sartipy F., Sigvant B., Lundin F., Wahlberg E. (2018). Ten Year Mortality in Different Peripheral Arterial Disease Stages: A Population Based Observational Study on Outcome. Eur. J. Vasc. Endovasc. Surg..

[19] Sebastianski M., Narasimhan S., Graham M.M., Toleva O., Shavadia J., Abualnaja S., Tsuyuki R.T., McMurtry M.S. (2014). Usefulness of the Ankle-Brachial Index to Predict High Coronary SYNTAX Scores, Myocardium at Risk, and Incomplete Coronary Revascularization. Am. J. Cardiol..

[20] Erkan H., Vatan B., Ağaç M.T., Korkmaz L., Erkan M., Kırış A., Acar Z., Çelik S. (2013). Relationship between SYNTAX score and Trans-Atlantic Inter-Society Consensus II classification in patients undergoing diagnostic angiography. Adv. Interv. Cardiol..

[21] Ralapanawa U., Sivakanesan R. (2021). Epidemiology and the Magnitude of Coronary Artery Disease and Acute Coronary Syndrome: A Narrative Review. J. Epidemiol. Glob. Health.

[22] Sanchis-Gomar F., Perez-Quilis C., Leischik R., Lucia A. (2016). Epidemiology of coronary heart disease and acute coronary syndrome. Ann. Transl. Med..

[23] Rafie A.H.S., Stefanick M.L., Sims S.T., Phan T., Higgins M., Gabriel A., Assimes T., Narasimhan B., Nead K.T., Myers J. (2010). Sex differences in the prevalence of peripheral artery disease in patients undergoing coronary catheterization. Vasc. Med..

[24] Farooq V., van Klaveren D., Steyerberg E.W., Meliga E., Vergouwe Y., Chieffo A., Kappetein A.P., Colombo A., Holmes D.R., Mack M. (2013). Anatomical and clinical characteristics to guide decision making between coronary artery bypass surgery and percutaneous coronary intervention for individual patients: Development and validation of SYNTAX score II. Lancet.

[25] Kobo O., Contractor T., Mohamed M.O., Parwani P., Paul T.K., Ghosh R.K., Alraes M.C., Patel B., Osman M., Ludwig J. (2020). Impact of pre-existent vascular and poly-vascular disease on acute myocardial infarction management and outcomes: An analysis of 2 million patients from the National Inpatient Sample. Int. J. Cardiol..

[26] Bashar H., Matetić A., Curzen N., Mamas M.A. (2022). Impact of extracardiac vascular disease on outcomes of 1.4 million patients undergoing percutaneous coronary intervention. Catheter. Cardiovasc. Interv..

[27] de Weerd M., Greving J.P., Hedblad B., Lorenz M.W., Mathiesen E.B., O’Leary D.H., Rosvall M., Sitzer M., Buskens E., Bots M.L. (2010). Prevalence of Asymptomatic Carotid Artery Stenosis in the General Population. Stroke.

[28] Donkor E.S. (2018). Stroke in the21stCentury: A Snapshot of the Burden, Epidemiology, and Quality of Life. Stroke Res. Treat..

[29] Li Z., Yang H., Zhang W., Wang J., Zhao Y., Cheng J. (2021). Prevalence of asymptomatic carotid artery stenosis in Chinese patients with lower extremity peripheral arterial disease: A cross-sectional study on 653 patients. BMJ Open.

[30] Topakian R., Nanz S., Rohrbacher B., Koppensteiner R., Aichner F.T. (2010). High Prevalence of Peripheral Arterial Disease in Patients with Acute Ischaemic Stroke. Cerebrovasc. Dis..

[31] Serena J., Segura T., Roquer J., García-Gil M., Castillo J., on behalf of the ARTICO Study (2015). The ARTICO study: Identification of patients at high risk of vascular recurrence after a first non-cardioembolic stroke. BMC Neurol..

[32] Wong N.D. (2020). Evaluating Multisite Atherosclerosis and its Progression. J. Am. Coll. Cardiol..

[33] Collet J.-P., Cayla G., Ennezat P.-V., Leclercq F., Cuisset T., Elhadad S., Henry P., Belle L., Cohen A., Silvain J. (2018). Systematic detection of polyvascular disease combined with aggressive secondary prevention in patients presenting with severe coronary artery disease: The randomized AMERICA Study. Int. J. Cardiol..

[34] Vasan R.S., Song R.J., Xanthakis V., Beiser A., DeCarli C., Mitchell G.F., Seshadri S. (2022). Hypertension-Mediated Organ Damage: Prevalence, Correlates, and Prognosis in the Community. Hypertension.

[35] Stone K., Fryer S., Faulkner J., Meyer M.L., Heffernan K., Kucharska-Newton A., Zieff G., Paterson C., Matsushita K., Hughes T.M. (2022). Associations of lower-limb atherosclerosis and arteriosclerosis with cardiovascular risk factors and disease in older adults: The Atherosclerosis Risk in Communities (ARIC) study. Atherosclerosis.

[36] Knuuti J., Wijns W., Saraste A., Capodanno D., Barbato E., Funck-Brentano C., Prescott E., Storey R.F., Deaton C., Cuisset T. (2020). 2019 ESC Guidelines for the diagnosis and management of chronic coronary syndromes. Eur. Heart J..

[37] López-Laguna N., Martínez-González M.A., Toledo E., Babio N., Sorlí J.V., Ros E., Muñoz M., Estruch R., Lapetra J., Muñoz-Bravo C. (2018). Risk of peripheral artery disease according to a healthy lifestyle score: The PREDIMED study. Atherosclerosis.

[38] Kraus W.E., Powell K.E., Haskell W.L., Janz K.F., Campbell W.W., Jakicic J.M., Troiano R.P., Sprow K., Torres A., Piercy K.L. (2019). Physical Activity, All-Cause and Cardiovascular Mortality, and Cardiovascular Disease. Med. Sci. Sports Exerc..

[39] Jansen S.C., Hoeks S.E., Nyklíček I., Scheltinga M.R., Teijink J.A., Rouwet E.V. (2022). Supervised Exercise Therapy is Effective for Patients with Intermittent Claudication Regardless of Psychological Constructs. Eur. J. Vasc. Endovasc. Surg..

[40] Wang W., Zhao T., Geng K., Yuan G., Chen Y., Xu Y. (2021). Smoking and the Pathophysiology of Peripheral Artery Disease. Front. Cardiovasc. Med..

[41] Creager M.A., Hamburg N.M. (2022). Smoking Cessation Improves Outcomes in Patients with Peripheral Artery Disease. JAMA Cardiol..

[42] Sharath S.E., Lee M., Kougias P., Taylor W.C., Zamani N., Barshes N.R. (2019). Successful Smoking Cessation Associated with Walking Behavior in Patients with Claudication. Ann. Vasc. Surg..

[43] Bragg F., Holmes M.V., Iona A., Guo Y., Du H., Chen Y., Bian Z., Yang L., Herrington W., Bennett D. (2017). Association between Diabetes and Cause-Specific Mortality in Rural and Urban Areas of China. JAMA.

[44] Barnes J.A., Eid M.A., Creager M.A., Goodney P.P. (2020). Epidemiology and Risk of Amputation in Patients with Diabetes Mellitus and Peripheral Artery Disease. Arter. Thromb. Vasc. Biol..

[45] Holman R.R., Paul S.K., Bethel M.A., Matthews D.R., Neil H.A. (2008). 10-year follow-up of intensive glucose control in type 2 diabetes. N. Engl. J. Med..

[46] Gerstein H.C., Miller M.E., Byington R.P., Goff D.C., Bigger J.T., Buse J.B., Cushman W.C., Genuth S., Ismail-Beigi F., Action to Control Cardiovascular Risk in Diabetes Study Group (2008). Effects of intensive glucose lowering in type 2 diabetes. N. Engl. J. Med..

[47] ADVANCE Collaborative Group (2008). Intensive blood glucose control and vascular outcomes in patients with type 2 diabetes. N. Engl. J. Med..

[48] Skeik N., Elejla S.A., Sethi A., Manunga J., Mirza A. (2023). Effects of SGLT2 inhibitors and GLP1-receptor agonists on cardiovascular and limb events in peripheral artery disease: A review. Vasc. Med..

[49] Zinman B., Wanner C., Lachin J.M., Fitchett D., Bluhmki E., Hantel S., Mattheus M., Devins T., Johansen O.E., Woerle H.J. (2015). Empagliflozin, Cardiovascular Outcomes, and Mortality in Type 2 Diabetes. N. Engl. J. Med..

[50] Bonaca M.P., Wiviott S.D., Zelniker T.A., Mosenzon O., Bhatt D.L., Leiter L.A., McGuire D.K., Goodrich E.L., De Mendonca Furtado R.H., Wilding J.P. (2020). Dapagliflozin and Cardiac, Kidney, and Limb Outcomes in Patients with and without Peripheral Artery Disease in DECLARE-TIMI 58. Circulation.

[51] Shah S.R., Najim N.I., Abbasi Z., Fatima M., Jangda A.A., Shahnawaz W. (2018). Canagliflozin and Cardiovascular disease-results of the CANVAS trial. J. Community Hosp. Intern. Med. Perspect..

[52] Perkovic V., Jardine M.J., Neal B., Bompoint S., Heerspink H.J.L., Charytan D.M., Edwards R., Agarwal R., Bakris G., Bull S. (2019). Canagliflozin and Renal Outcomes in Type 2 Diabetes and Nephropathy. N. Engl. J. Med..

[53] Caruso P., Maiorino M.I., Bellastella G., Esposito K., Giugliano D. (2023). Pleiotropic effects of GLP-1 receptor agonists on peripheral artery disease: Is there any hope?. Diabetes/Metab. Res. Rev..

[54] Dhatariya K., Bain S.C., Buse J.B., Simpson R., Tarnow L., Kaltoft M.S., Stellfeld M., Tornøe K., Pratley R.E., the LEADER Publication Committee on behalf of the LEADER Trial Investigators (2018). The Impact of Liraglutide on Diabetes-Related Foot Ulceration and Associated Complications in Patients with Type 2 Diabetes at High Risk for Cardiovascular Events: Results from the LEADER Trial. Diabetes Care.

[55] Lin D.S.-H., Lee J.-K., Chen W.-J. (2021). Major adverse cardiovascular and limb events in patients with diabetes treated with GLP-1 receptor agonists vs. DPP-4 inhibitors. Diabetologia.

[56] Scheen A.J. (2022). Lower limb amputations: Protection with GLP-1 receptor agonists rather than increased risk with SGLT2 inhibitors?. Diabetes Metab..

[57] Caruso I., Cignarelli A., Sorice G.P., Natalicchio A., Perrini S., Laviola L., Giorgino F. (2022). Cardiovascular and Renal Effectiveness of GLP-1 Receptor Agonists vs. Other Glucose-Lowering Drugs in Type 2 Diabetes: A Systematic Review and Meta-Analysis of Real-World Studies. Metabolites.

[58] Du Y., Bai L., Fan B., Ding H., Ding H., Hou L., Ma H., Xing N., Wang F. (2022). Effect of SGLT2 inhibitors versus DPP4 inhibitors or GLP-1 agonists on diabetic foot-related extremity amputation in patients with T2DM: A meta-analysis. Prim. Care Diabetes.

[59] Cosentino F., Grant P., Aboyans V., Bailey C.J., Ceriello A., Delgado V. (2020). The Task Force for diabetes, pre-diabetes, and cardiovascular diseases of the European Society of Cardiology (ESC) and the European Association for the Study of Diabetes (EASD). Eur. Heart J..

[60] Emdin C., Anderson S., Callender T., Conrad N., Salimi-Khorshidi G., Mohseni H., Woodward M., Rahimi K. (2015). Usual blood pressure, peripheral arterial disease, and vascular risk: Cohort study of 4.2 million adults. BMJ.

[61] Williams B., Mancia G., Spiering W., Agabiti Rosei E., Azizi M., Burnier M., Clement D.L., Coca A., de Simone G., Dominiczak A. (2018). 2018 ESC/ESH Guidelines for the management of arterial hypertension. Eur. Heart J..

[62] Östergren J., Sleight P., Dagenais G., Danisa K., Bosch J., Qilong Y., Yusuf S. (2004). Impact of ramipril in patients with evidence of clinical or subclinical peripheral arterial disease. Eur. Heart J..

[63] Heagerty A., Yusuf S., Teo K.K., Pogue J., Dyal L., Copland I., Schumacher H., Dagenais G., Sleight P., Anderson C. (2008). Telmisartan, Ramipril, or Both in Patients at High Risk for Vascular Events. N. Engl. J. Med..

[64] Shahin Y., Barnes R., Barakat H., Chetter I.C. (2013). Meta-analysis of angiotensin converting enzyme inhibitors effect on walking ability and ankle brachial pressure index in patients with intermittent claudication. Atherosclerosis.

[65] Khan S.Z., O’Brien-Irr M.S., Rivero M., Blochle R., Cherr G.S., Dryjski M.L., Dosluoglu H.H., Lukan J., Rowe V.L., Harris L.M. (2020). Improved survival with angiotensin-converting enzyme inhibitors and angiotensin receptor blockers in chronic limb-threatening ischemia. J. Vasc. Surg..

[66] Radack K., Deck C. (1991). Beta-adrenergic blocker therapy does not worsen intermittent claudication in subjects with peripheral arterial disease. A meta-analysis of randomized controlled trials. Arch. Intern. Med..

[67] Zeitouni M., Sabouret P., Kerneis M., Silvain J., Collet J.-P., Bruckert E., Montalescot G. (2020). 2019 ESC/EAS Guidelines for management of dyslipidaemia: Strengths and limitations. Eur. Heart J.—Cardiovasc. Pharmacother..

[68] Belur A.D., Shah A.J., Virani S.S., Vorla M., Kalra D.K. (2021). Role of Lipid-Lowering Therapy in Peripheral Artery Disease. J. Clin. Med..

[69] (2007). Heart Protection Study Collaborative Group Randomized trial of the effects of cholesterol-lowering with simvastatin on peripheral vascular and other major vascular outcomes in 20,536 people with peripheral arterial disease and other high-risk conditions. J. Vasc. Surg..

[70] Pastori D., Farcomeni A., Milanese A., Del Sole F., Menichelli D., Hiatt W.R., Violi F. (2020). Statins and Major Adverse Limb Events in Patients with Peripheral Artery Disease: A Systematic Review and Meta-Analysis. Thromb. Haemost..

[71] Cannon C.P., Blazing M.A., Giugliano R.P., McCagg A., White J.A., Théroux P., Darius H., Lewis B.S., Ophuis T.O., Jukema J.W. (2015). Ezetimibe Added to Statin Therapy after Acute Coronary Syndromes. N. Engl. J. Med..

[72] Bonaca M.P., Gutierrez J.A., Cannon C., Giugliano R., Blazing M., Park J.-G., White J., Tershakovec A., Braunwald E. (2018). Polyvascular disease, type 2 diabetes, and long-term vascular risk: A secondary analysis of the IMPROVE-IT trial. Lancet Diabetes Endocrinol..

[73] Bonaca M.P., Nault P., Giugliano R.P., Keech A.C., Pineda A.L., Kanevsky E., Kuder J., Murphy S.A., Jukema J.W., Lewis B.S. (2018). Low-Density Lipoprotein Cholesterol Lowering with Evolocumab and Outcomes in Patients with Peripheral Artery Disease: Insights from the FOURIER Trial (Further Cardiovascular Outcomes Research with PCSK9 Inhibition in Subjects with Elevated Risk). Circulation.

[74] Jukema J.W., Szarek M., Zijlstra L.E., de Silva H.A., Bhatt D.L., Bittner V.A., Diaz R., Edelberg J.M., Goodman S.G., Hanotin C. (2019). Alirocumab in Patients with Polyvascular Disease and Recent Acute Coronary Syndrome: ODYSSEY OUTCOMES Trial. J. Am. Coll. Cardiol..

[75] Ray K.K., Troquay R.P.T., Visseren F.L.J., Leiter L.A., Wright R.S., Vikarunnessa S., Talloczy Z., Zang X., Maheux P., Lesogor A. (2023). Long-term efficacy and safety of inclisiran in patients with high cardiovascular risk and elevated LDL cholesterol (ORION-3): Results from the 4-year open-label extension of the ORION-1 trial. Lancet Diabetes Endocrinol..

[76] Seijas-Amigo J.P., Cordero A., del Olmo F., Quiroga G.A.C., Fácila L., Salgado-Barreira P., Reyes-Santías F., Romero-Menor C., Murillo J.R., Rodríguez-Mañero M. (2023). Patients with High Cardiovascular Risk as Candidates to Bempedoic Acid, after Treatment with Statins, Ezetimibe and PCSK9 Inhibitors: An Estimation and Cost-Effectiveness Analysis. J. Cardiovasc. Pharmacol..

[77] Belch J., MacCuish A., Campbell I., Cobbe S., Taylor R., Prescott R., Lee R., Bancroft J., MacEwan S., Shepherd J. (2008). The prevention of progression of arterial disease and diabetes (POPADAD) trial: Factorial randomised placebo controlled trial of aspirin and antioxidants in patients with diabetes and asymptomatic peripheral arterial disease. BMJ.

[78] Fowkes F.G.R., Price J.F., Stewart M.C.W., Butcher I., Leng G.C., Pell A.C.H., Sandercock P.A.G., Fox K.A.A., Lowe G.D.O., Murray G.D. (2010). Aspirin for Prevention of Cardiovascular Events in a General Population Screened for a Low Ankle Brachial IndexA Randomized Controlled Trial. JAMA.

[79] Creager M.A. (1998). Results of the CAPRIE trial: Efficacy and safety of clopidogrel. Vasc. Med..

[80] Hiatt W.R., Fowkes F.G.R., Heizer G., Berger J.S., Baumgartner I., Held P., Katona B.G., Mahaffey K.W., Norgren L., Jones W.S. (2017). Ticagrelor versus Clopidogrel in Symptomatic Peripheral Artery Disease. N. Engl. J. Med..

[81] Bhatt D.L., Fox K.A., Hacke W., Berger P.B., Black H.R., Boden W.E., Cacoub P., Cohen E.A., Creager M.A., Easton J.D. (2006). Clopidogrel and Aspirin versus Aspirin Alone for the Prevention of Atherothrombotic Events. N. Engl. J. Med..

[82] Aboyans V., Bauersachs R., Mazzolai L., Brodmann M., Palomares J.F.R., Debus S., Collet J.-P., Drexel H., Espinola-Klein C., Lewis B.S. (2021). Antithrombotic therapies in aortic and peripheral arterial diseases in 2021: A consensus document from the ESC working group on aorta and peripheral vascular diseases, the ESC working group on thrombosis, and the ESC working group on cardiovascular pharmacotherapy. Eur. Heart J..

[83] Eikelboom J.W., Connolly S.J., Bosch J., Dagenais G.R., Hart R.G., Shestakovska O., Diaz R., Alings M., Lonn E.M., Anand S.S. (2017). Rivaroxaban with or without Aspirin in Stable Cardiovascular Disease. N. Engl. J. Med..

[84] Tepe G., Bantleon R., Brechtel K.F.M., Schmehl J.-M., Zeller T., Claussen C.D., Strobl F.F.X. (2012). Management of peripheral arterial interventions with mono or dual antiplatelet therapy—The MIRROR study: A randomised and double-blinded clinical trial. Eur. Radiol..

[85] Dutch Bypass Oral Anticoagulants or Aspirin (BOA) Study Group (2000). Efficacy of oral anticoagulants compared with aspirin after infrainguinal bypass surgery (The Dutch Bypass Oral Anticoagulants or Aspirin Study): A randomised trial. Lancet.

[86] Belch J.J.F., Dormandy J., Biasi G.M., Cairols M., Diehm C., Eikelboom B., Golledge J., Jawien A., Lepäntalo M., CASPAR Writing Committee (2010). Results of the randomized, placebo-controlled clopidogrel and acetylsalicylic acid in bypass surgery for peripheral arterial disease (CASPAR) trial. J. Vasc. Surg..

[87] Bonaca M.P., Bauersachs R.M., Anand S.S., Debus E.S., Nehler M.R., Patel M.R., Fanelli F., Capell W.H., Diao L., Jaeger N. (2020). Rivaroxaban in Peripheral Artery Disease after Revascularization. N. Engl. J. Med..

[88] Hiatt W.R., Bonaca M.P., Patel M.R., Nehler M.R., Debus E.S., Anand S.S., Capell W.H., Brackin T., Jaeger N., Hess C.N. (2020). Rivaroxaban and Aspirin in Peripheral Artery Disease Lower Extremity Revascularization: Impact of Concomitant Clopidogrel on Efficacy and Safety. Circulation. Circulation.

[89] Argyriou C., Saleptsis V., Koutsias S., Giannoukas A.D. (2013). Peripheral Arterial Disease Is Prevalent But Underdiagnosed and Undertreated in the Primary Care Setting in Central Greece. Angiology.

[90] Saleh A., Makhamreh H., Qoussoos T., Alawwa I., Alsmady M., Salah Z.A., Shakhatreh A., Alhazaymeh L., Jabber M. (2018). Prevalence of previously unrecognized peripheral arterial disease in patients undergoing coronary angiography. Medicine.

[91] Saxon J.T., Safley D.M., Mena-Hurtado C., Heyligers J., Fitridge R., Shishehbor M., Spertus J.A., Gosch K., Patel M.R., Smolderen K.G. (2020). Adherence to Guideline-Recommended Therapy—Including Supervised Exercise Therapy Referral—Across Peripheral Artery Disease Specialty Clinics: Insights from the International PORTRAIT Registry. J. Am. Heart Assoc..

[92] Armstrong E.J., Chen D.C., Westin G.G., Singh S., McCoach C.E., Bang H., Yeo K., Anderson D., Amsterdam E.A., Laird J.R. (2014). Adherence to Guideline-Recommended Therapy Is Associated with Decreased Major Adverse Cardiovascular Events and Major Adverse Limb Events Among Patients with Peripheral Arterial Disease. J. Am. Heart Assoc..

[93] Smolderen K.G., Romain G., Provance J.B., Scierka L.E., Mao J., Goodney P.P., Henke P.K., Sedrakyan A., Mena-Hurtado C. (2023). Guideline-Directed Medical Therapy and Long-Term Mortality and Amputation Outcomes in Patients Undergoing Peripheral Vascular Interventions. JACC Cardiovasc. Interv..

